# Initial posting—a critical stage in the employment cycle: lessons from the experience of government doctors in Gujarat, India

**DOI:** 10.1186/s12960-016-0138-3

**Published:** 2016-07-11

**Authors:** Bhaskar Purohit, Tim Martineau

**Affiliations:** Indian Institute of Public Health Gandhinagar (IIPHG), Sardar Patel Institute Campus, Drive in Road, Thaltej, Ahmedabad, 380054 India; Liverpool School of Tropical Medicine (LSTM), Pembroke Place, Liverpool, L3 5QA United Kingdom

**Keywords:** Personnel management, Doctor, Public sector, Posting and transfer, India

## Abstract

**Background:**

With the critical shortage of government doctors serving in rural health centers in India, understanding the initial posting policies, processes, and practices become important from a retention point of view. The initial posting is a very critical stage of an employment cycle and could play an important role in influencing the key human resource for health outcomes such as turnover and performance. The current study aimed at exploring a rather unknown phenomenon of the initial posting-related processes, practices, and perceptions of Medical Officers working with the Public Health Department in Gujarat, India.

**Methods:**

This was an exploratory study carried out in the state of Gujarat, India, that used qualitative methods first to document the extant initial posting policy with the help of document review and five Key Informant interviews; next, 19 in-depth interviews were carried out with Medical Officers to assess implementation of policies as well as processes and systems related to the initial posting of Medical Officers. A thematic framework approach was used to analyze qualitative data using NVIVO.

**Results:**

The results indicate that there is no formal published or written initial posting policy in the state, and in the absence of a written and formal policy, the overall posting systems were perceived to be arbitrary by the study respondents. In the absence of any policy, the state has some unwritten informal practices such as posting the Medical Officers at their native places. Although this practice reflects a concern towards the Medical Officer’s needs, such practices are not consistently applied indicating some inequity and possible implications over Medical Officers’ retention and motivation.

**Conclusions:**

Initial posting is a critical aspect of an employment cycle, and the perceptions and experiences of MOs regarding the processes and practices involved in their initial posting can be crucial in influencing their performance and turnover rates. If long-term solutions are to be sought in addressing the availability and distribution of Medical Officers in the state, then there is a need to have clearly laid down initial posting-related policies that reflect the equity and consideration towards Medical Officers in placement-related matters.

## Background

Shortage and maldistribution of the health workforce is a global phenomenon. Such shortage and maldistribution is particularly pronounced for highly skilled health service providers such as doctors and nurses who are also the frontline managers [1]. The issue of shortage and maldistribution is important as it is linked to the poor coverage of basic health services in several countries [1, 2] and poor quality of care being provided to people [3]. Although doctors are regarded as the frontline managers and are key to providing care, India faces a severe shortage of doctors or Medical Officers (MOs) working with the government sector, particularly in rural areas in India. There is also a considerable imbalance in where and how the MOs are placed and distributed with imbalances existing between the states as well as within the states and most of the concentration of MOs in urban areas in India [4].

Initial posting or placement has been identified as critical as most of the turnover in organizations happen during the initial few months of starting a job. During the initial few months of a job, new recruits may develop negative perceptions about the job particularly because of absence of any induction process in place or as a result of the perception of being placed in a position where their skills are not effectively utilized [5]. Hence, the initial posting or placement policies, processes, and practices become very important from a retention point of view. The experience of initial posting is very important, and a positive experience with the same can go a long way leading to positive organizational commitment [6]. Research with graduate students in public health from India suggests that placement can be an important motivational factor and inappropriate placements can negatively influence the ability of the recruits to apply their learning in their respective workplaces [7]. Human resource management (HRM) has been recognized as the major factor in encouraging organizational innovation and performance [8, 9], and the key to achieving these goals is through the implementation of HRM policies that attract, develop, and retain the best talent. Hence, this paper focuses on a critical aspect of HRM—initial posting—that not only has the potential to attract MOs.

**Fig. 1:**
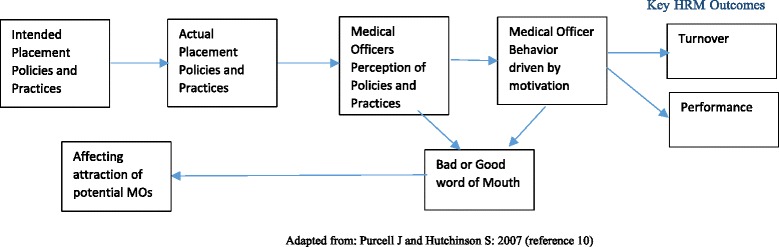
Conceptual framework for the study: initial posting—a critical stage in the employment cycle

Figure 1 (adapted from Purcell and Hutchinson [10]) shows the conceptual framework used for the study. The initial posting is a very critical stage of an employment cycle and could play an important role in influencing the key human resource for health (HRH) outcomes such as turnover and performance. The health department in the effort of better HRH management may have certain intended initial posting-related policies (written or unwritten) and practices. These intended policies and practices may further guide the actual policies and practices; however, there may be a gap between intended and actual practices. The actual processes and practices then play a crucial role in shaping the positive or negative perceptions MOs have about posting-related processes and practices. The negative or positive perceptions depend upon how the actual policies and practices are implemented on an individual basis as the MOs tend to compare the benefits or treatments they have received compared to their counterparts. The equity theory of motivation suggests that perceptions people have about how they are being treated or rewarded as compared with others have influence over their motivations [11]. The MOs’ perceptions depending on whether they are positive or negative further affect the MOs’ motivation (positively or negatively) having an influence over their behavior leading to either good or poor performance or the decisions to continue with their current job that affects the turnover. If the MOs have positive perceptions about the policies, processes, and practices, then it may influence their behavior positively having a favorable impact on HRH-related outcomes such as better performance and reduced turnover and vice versa (see Fig. 1 for details).

The process of initial posting may also affect the attraction towards the job positively or negatively. This may be contingent upon the perceptions and experiences of MOs (who are already in the service) with initial postings and how such perceptions and experiences through bad or good word of mouth may influence the decisions of MOs who contemplate to join the government in the future.

Although initial posting is an important aspect of an employment cycle, it has rarely been studied in the public health sector in India. Understanding the initial posting policies, processes, and practices in the public health sector becomes particularly important because of the critical shortage of MOs in the public health sector and the preference of medical graduates to join the private sector instead. The HRM systems in the public health sector can often be slow, ineffective, and unresponsive to HRH needs, and as a result, such systems run the risk of further losing the limited MOs who want to join the government affecting attraction or losing the ones who have already joined through voluntary turnover. For example, evidence from India [12] and other countries suggests that delays in public systems relating to HRM functions such as slow recruitment can lead to poor motivation and turnovers [13, 14]. Similarly, the issues with inappropriate postings and transfers can be a cause of low morale, geographical maldistribution, and migration of MOs [15]. Further, poor posting and transfer policies and practices directly or indirectly prevent or discourage health care providers from either joining the public health sector or such policies contribute to the provider’s dissatisfaction with the existing system and may lead to low morale, poor performance, high absenteeism, and attrition [16, 17]. Hence, addressing the HRH-related policies and systems can play a major role in influencing retention and possibly attraction too—positively.

With this backdrop, the current study focused on exploring the policies, processes, and practices relating to initial posting for MOs working with a state-level government health department in India. As no studies were found that investigated initial posting-related policies, processes, and systems for government doctors in India, the study looks at a rather unknown yet crucial phenomenon for the effective management of HRH which could potentially ensure adequate and equitable staffing across the services and locations while minimizing the negative impact on staff motivation.

Gujarat State was selected for this study as it represents one of the most economically progressive states of India with health indicators better than the national average, yet the state suffers from shortage of MOs and specialists, especially in rural areas [18]. The vacancy and shortfall in the state is 24 % for MOs at primary health centers (PHCs) while the vacancy and shortfall is particularly high (77 and 93 %, respectively) for all specialists working with community health centers (CHCs) [18]. Another reason for selecting Gujarat was the presence of a public health institute in the state called the Indian Institute of Public Health Gandhinagar (IIPHG) that is working closely with the Department of Health, Government of Gujarat, to strengthen the health system in the state. The selection of Gujarat not only served the mandate of IIPHG to strengthen the health system in the state through research but seeking permission for the study from the state was also easier.

While the production of doctors in Gujarat has been sufficient to meet the shortages, very few medical graduates from the state actually join the government service which makes addressing the shortage of doctors a complicated issue. The state of Gujarat has compulsory rural practice for government college medical graduates called “bonded doctors.” Paradoxically, despite producing many medical graduates every year [19, 20], very few actually join the government service [21]. For example, 50 % of MOs appointed on ad hoc or temporary basis actually joined the government in 2004–2005 and 2005–2006 [22].

### The health system in Gujarat

As per the state’s Civil Services Recruitment Rules 1967, the MOs have been categorized into two classes: I and II. Both classes I and II are gazetted posts, and the state’s Public Service Commission (PSC) called the Gujarat Public Service Commission (GPSC) is responsible for the recruitment of all gazetted posts including MOs [23]. All the graduate doctors are recruited as Medical Officers (MOs) in class II whereas those holding post-graduate degree in clinical areas are recruited as specialist class I. All specialist and senior positions at district and state levels are class I positions while the MOs working with PHCs and CHCs without post-graduate specialization are class II positions. According to health service norms, each CHC needs to be staffed with specialists as well as regular doctors or MOs. Similarly, each PHC needs to be staffed with at least one MO.

At the district level, the Chief District Health Officer (CDHO) who is a class I officer is overall in charge of the CHCs and PHCs within the district. *Several blocks or the administrative units constitute a district*. Blocks are administered by the Block Health Officers (BHOs) which is also a class II position.

#### Bonded MOs

Under the compulsory rural service in Gujarat, all the medical graduates from the government colleges enter the government service under the “bonded” category and are required to sign a bond at the time of admission to medical college that requires them to compulsorily serve in rural areas for 2 years.

#### Ad hoc MOs

To address the shortage of MOs in the state, the Department of Health and Family Welfare in the past recruited MOs from Gujarat such as candidates from private medical colleges or outside the state. Recruitment of such MOs is called ad hoc appointment. MOs under ad hoc appointment were appointed on a temporary basis and are required to pass the states’ Public Service Commission (PSC) exam called GPSC in order to be appointed as permanent employees which would give them regular service.

#### Contractual MOs

Yet another category under which MOs are recruited is called “contractual appointment” where the appointments are done for 11 months. The contractual category includes all the MOs from private medical colleges or from an outside state or the ones who do not meet the age criterion of the GPSC but who wish to work with the government health department. Contractual MOs may also include MOs from alternative systems such as homeopathy or Ayurveda. The MOs under contractual category do not get employment benefits which are otherwise available to MOs who are on “regular service” till the time they pass the states’ PSC exam.

#### District hospital (DH)

DH is a public hospital that caters to the health needs of the entire district providing mainly tertiary care.

#### Community health center (CHC)

The CHC is a 30-bed hospital that constitutes the secondary level of health care and provides referral as well as specialist health care to the rural population at the block level. It caters to 80 000–120 000 population.

#### Primary health center (PHC)

The PHC covers a population of 20 000 in hilly, tribal, or difficult areas and 30 000 populations in plain areas with four to six indoor/observation beds. It acts as a referral unit for six sub-centers and refers out cases to CHC (30-bed hospital) and higher order public hospitals located at the sub-district and district levels.

## Methods

### Study design

This was a qualitative study that mainly used in-depth interviews with 19 MOs and five Key Informants. To document the extant initial posting policy, document review and five in-depth interviews with Key Informants were conducted; next, 19 in-depth interviews were carried out with MOs to obtain an in-depth understanding of the processes and systems related to initial posting of MOs in order to assess implementation of policies. The study used in-depth qualitative methods as it was best suited to the scope of the current study that aimed at exploring a rather unknown phenomenon of the initial posting-related processes, practices, and perceptions of MOs that would not have been possible through quantitative study. Qualitative design also justifies the need for the study aimed at organizing the data into themes presented in the “Results” section [24].

### Study setting

This study was conducted in Gujarat, India, in 2013. MOs were included as the main respondents of the study working for the government health department placed at rural health centers from three different districts from the state. Based on initial discussions with several MOs and state-level officers prior to the study, a list of a few “desirable,” “not so desirable,” and “not at all desirable” districts for MO posting was made. The main criterion for “desirable,” “not so desirable,” and “not at all desirable” districts was mainly doctors’ willingness in general to be posted to such districts. The doctors’ willingness to be posted is influenced by factors such as close proximity to state health headquarters and easy access (desirable district) compared to hard to reach districts (not at all desirable districts) that are sometimes far from state headquarters. As several districts were identified in each of the above category, three districts meeting the above criteria were selected from the different regions from the state for larger geographical representation.

### Data collection methods and sampling

#### Document review

Document review was carried out to understand the extant placement-related rules and policies. Under document reviews, several documents such as recruitment rules, government orders, transfer guidelines, and appointment orders were reviewed.

#### Interviews with Key Informants

This group comprised of informants who occupied key state- and district-level positions purposively selected for their knowledge of the study topic. The study guide included questions to gauge their opinions on the existing policies and how policies are implemented. The study included five Key Informants to ensure that the views and perspectives of the range of stakeholders on the study topic could be represented. This included two current and one retired senior state-level official from the health department, a representative from the administrative department, and a representative from the Medical Association of Gujarat. Three out of five interviews were conducted in Hindi, and two Key Informants preferred to be interviewed in English using topic guides. All the Key Informant interviews were captured using a digital recorder.

#### Interview with MOs

This group consisted of class I and II MOs who were the main subjects of the study. MOs working with only PHCs and CHCs and those from block level were included in the study. The study guide included questions to assess MOs’ perceptions and understanding and their experiences of initial posting- or placement-related processes and systems. All the interviews were recorded digitally.

#### Sampling technique for MOs

The study used purposive sampling at various stages while selecting the study respondents. This purposive selection approach focused on ensuring representation of both male and female doctors; those with medical graduate degree and/or post-graduate medical degree and doctors from block level; and regular MOs that were Gujarat Public Service Commission (GPSC) confirmed from different geographical locations within the state. The total number of interviews with MOs was conducted till the time saturation in information was experienced. In total, 19 in-depth interviews were done with the MOs in Hindi. The main objective to interview MOs was to explore MOs’ perceptions, understanding, and experiences of placement-related policies and systems. The study tried to include both male and female respondents; however, due to the overall shortage of female MOs in the state, a total 16 respondents were males while only three were females.

### Data analysis

#### Document review analysis

Simple content analysis of the documents was done to understand the existing placement-related policies [25].

#### In-depth interviews and analysis

All interview recordings were transcribed verbatim and then translated into English text. Interviews were analyzed using a thematic framework approach which is a matrix-based method to arrange and synthesize data [24]. The framework analysis approach was best suited to the scope of current research as the aim of the research was to present themes identified in the data. To analyze the data, study objectives, interview guide, and methodology adopted were regularly revisited. A framework approach was used to identify key words, themes, and sub-themes, and the transcripts of the 24 participants (Key Informants and MOs) were coded and grouped according to the themes and sub-themes identified. A detailed analysis was performed using NVIVO on the transcribed texts.

As the aim of the research was to present themes identified in the data, the study identified several themes presented in the form of the following questions explained in details in the “Results” section.

1. Current initial placement-related policies
2. Choice of location while posting MOs
3. Basis of posting
4. Consistency in application of policies and practices
5. Issues in placement-related processes and practices
6. Recommendations from MOs to improve the processes and practices

#### Research ethics

Due to the sensitive nature of the study topic that involved interviews with MOs and Key Informants, the study took great care in maintaining anonymity of the respondents. Written consent was sought from study participants before audio recording of the interviews. The ethical approval for the study was sought from the institutional ethical review committee at Indian Institute of Public Health Gandhinagar (IIPHG). Relevant permission for the study was also obtained from the Department of Health, Government of Gujarat, and the Commissionerate of Health, Gujarat. Further, written consent was obtained from all the MOs and Key Informants. The participation in the study was completely voluntary, and respondents were assured of anonymity at all times of the study.

## Results

The demographic profile of the study respondents is presented in Table 1. Next in the “Results” section, the policies and practices relating to initial posting are presented in the following policy areas:

**Table 1:** Distribution of Medical Officers based on demographic- and work-related variables

	District 1	District 2	District 3	Total
Gazetted officer				
Class I	1	1	1	3
Class II	4	5	7	16
Gender				
Male	5	5	6	16
Female	0	1	2	3
Entered service through				
Bonded	3	4	4	11
Ad hoc	2	2	4	8
Place of work				
PHC	1	3	3	7
CHC	0	2	2	4
SDH/DH	3	1	0	4
BHO	1	0	3	4

1. Availability of data for planning placements
2. Preferences for initial placement and initial placement at native place
3. The administration of placement orders
4. Perceptions and possible implications of posting MOs at their native places
5. Suggestions from MOs to improve the placement processes

### Current policy and practices

The authors could not identify any formal published placement policy in the state. Interviews with Key Informants confirm that there is no written policy or a guideline that is followed in the state to initially place the bonded and ad hoc MOs at health centers. However, the document review as well as responses from Key Informants suggests that there is one transfer-related rule which also pertains to placement. This rule prohibits the gazetted officers from different departments being placed at their native place. However, class I and II officers from Health are exempt from this rule [26]. This shows some flexibility for the health department as far as the implementation of the abovementioned rule is concerned.

Both Key Informants and MOs suggested there was no clear rationale behind the initial placement of MOs. According to study respondents, the health department usually tries to place the bonded MOs at their native place or close to their native place. However, a few respondents reported that the placements are often very arbitrarily done by clerical staff.

For posting there are no guidelines and it depends on the person who is posting the MOs. …..According to the resolutions, no gazetted officer can be posted at their native place (according to the guideline of the state government). But against these guidelines MOs are exempted and in fact they are preferred in their native place. This is because if this preference is not given then MOs are not likely to join which can lead to lot of vacant posts. So considering this, state government has decided that MOs can be posted at their native place (Key Informant 1)

There is no definite clear cut guideline or policy how to declare various positions and how to place MOs at such positions (Key Informant 4)

The health department have their own way to decide. There is a clerk that sits there and decides haphazardly how these candidates would be posted (MO 2)

### Availability of data for planning placements

The study found that in order to ensure MOs’ availability across the state, the health department tries to place the MOs at their native place or close to their native place. The data for native place is made available to the health department from the list of graduates that is made available to the health department by the medical colleges.

The health department while placing a doctor takes into consideration the place MOs belong to. They generally try to place MOs at their native place but if the positions are 100 % full, then the MO will be placed in some other District which is nearby (MO 13)

MOs are given preference to work at their native places. This is because if this preference is not given then MOs are not likely to join which can lead to lot of vacant posts. So considering this, state government has decided that MOs can be posted at their native place (Key Informant 1)

### Preference for initial placement and initial placement at native place

#### Are MOs given any preference for their initial posting?

There are inconsistencies in the practices of initial posting of bonded MOs. In general, the health department uses no formal mechanisms for seeking the preferences for initial placements of bonded MOs though the study identified two exceptions to this practice.

Yes I gave my choice of posting and I was given my choice (MO 10)

No they did not ask for choice of location (MO 12)

#### Were bonded MOs posted at their native places?

Next, we assessed whether bonded MOs were actually placed at their native places or not. What can be concluded based on the responses of MOs is that while some of them were placed at their native places, there were others who were not even placed close to their native place. The study found that in a few cases, MOs used political contacts to get placed at desirable places.

Yes, definitely the Government gives placement to bonded candidates in their native place. I got my order in the last month of internship and my native place was Z so I was placed at Z district (MO 18)

In 80 % of the cases, people do not get placed or posting at their native place (MO 2)

The idea is, as I have heard that MOs are placed at their native places or close to their native place. Although the health department claims to do like this, they don’t do it. In my case it was not done… Let me tell you something. My first job order was for district X but I did not want to go there. I had to use my influence to get my posting elsewhere (MO 2)

#### Initial posting of ad hoc MOs

The study found little difference in the processes of initial placement for ad hoc doctors. Some were asked what their preferences were; others were not. Those that were asked did not necessarily get their choice because of lack of vacancies

There are walk-in- interviews held at Gandhinagar [the state capital]. I gave my three preferences as A, B, and C but they said that no place were available there so they placed me somewhere else (MO 7)

The health department did not ask for choice of location and hence I did not get my choice of location (MO 1)

### The administration of placement orders

The study respondents under the bonded category shared their experiences about the time it takes to receive placement orders from the government to the time it takes for initial posting. While most of the respondents were of the opinion that such orders are received timely (mostly during the last of month of their medical internship), a delay of up to 6 months was reported in one or two cases. However, administration of placement orders was found timely for MOs under ad hoc category.

As soon as the bonded MOs finish your internship, they must receive the placement orders. Such orders are received after 3-6 months after finishing the internship. If health department can send such orders just before the completion of internship, at least 5 % MOs would join. Since the health department sends it after 6 months, they are not even aware how many have MOs have enrolled for PG or even how many are dead or alive (MO 17)

### Perceptions and possible implications of posting MOs at their native places

According to MOs and Key Informants, placement of MOs at their preferred place has a bearing on MOs’ decisions to join and continue service with the government. The responses from Key Informants as well as the MOs indicate that placing MOs at their preferred place can lead to better work and performance from MOs. The study respondents further suggested that this can also lead to positive satisfaction among the MOs, especially among the contractual MOs, thereby reducing the turnover rates as indicated in the study framework presented in Fig. 1.

However, one of the respondents perceived the rationale of placing MOs at their native place to be an ineffective human resource strategy to address the shortage of MOs in the state as there are MOs who have aspirations to move from rural or tribal places to bigger cities.

Only If I am given native or desirable places, only then I will be interested in the job (MO 2)

I am telling you that the doctors who study around big cities even though their natives may be in some tribal area, they don’t want to go back to the tribal areas (MO 16)

The staff recruited on contractual basis may feel that they might be discontinued from services any time. Sometimes they don’t join because they are not given their preferred place of posting. For example- somebody interested in clinical work if posted at PHC may not like to continue his work at PHC because PHC work hardly involves clinical duties (MO 1)

First of all the department should inform about all the positions vacant in various places. If the contractual MOs feel they have been given one of his choice districts, only then they would do better work. And they should also feel confident that he will continue in that place for at least 3 yrs. It should not be that he is transferred in 9 months. If you don’t give them choice district then they may leave the service in 6 or 9 months etc or will not feel good about the posting (MO 5)

### Suggestions from MOs to improve the placement processes

Irrespective of the category of employment (bonded or ad hoc), respondents highlighted the need and importance of giving posting at preferred places, as this had an impact on their performance at work. A policy that assures that such placements are not erratic and will not disturb the candidate for 3 years can give more stability and confidence to MOs. One of the respondents also highlighted the importance of strategic placement by posting the MOs where they are needed most rather than following the facility staffing norms which do not necessarily reflect the actual staffing need.

They should ask. Every person is comfortable working in their own Taluka/District/State. So if one is asked and given his preferred location then they will be more stable there. There are many self-financed medical colleges also, many doctors pass out of those colleges. So anyone who works in his own place will work better. There are places where MOs don’t like to be posted (MO 3)

## Discussion

This was an exploratory study and also the first of its kind in the state that aimed to explore an unknown but important phenomenon—the initial posting-related policies, systems, and processes for MOs and its possible implications for improved availability and retention of MOs that could potentially address the shortage of MOs in the state of Gujarat, India. However, due to limited geographical focus and only 24 respondents included in the study, there is a need to explore the phenomenon of initial posting using larger studies to identify the scale of the problem at the state and national levels.

The practice of the health department of posting MOs at their native districts or close to their native district is not clearly guided by any formal state policy but rather guided by an implementation of unwritten rules. Such a practice nonetheless reflects good intention of the state, using the flexibility with public service regulations given to the health department, suggesting consideration towards MOs’ needs and interest. This practice is also in line with WHO recommendations for native placement for better retention [27], and similar practices have also been adopted in other countries such as Bangladesh where according to public medical college admission rules the applicants need to be selected based on the district they originally represent [28]. However, in the absence of any clear policies, the study results indicate non-uniform criteria and inconsistent practices for placing MOs where some were placed at their native districts while others were placed neither at native districts nor close to their native districts. Similar inconsistencies were also reported as far as opportunity for placement at choice of locations was concerned. Although the state has the well-intended HR practice to place MOs at native or close to native places, if staff feel that they are being unfairly treated, this may have a negative impact on their level of motivation, as explained by Equity theory [11]. This may lead to poor performance or voluntary turnover as indicated by the study respondents.

The current placement-related practices may also have an influence over the attraction of potential MOs to the government sector. With already a severe shortage of MOs in the government sector [18], with less than 10 % joining the government sector, bad or good experiences shared by word of mouth may play an important role in influencing the initial attraction of MOs to the service.

Though there are clear benefits, the idea of placing MOs at their native places has several problems. The first problem is that despite having good intentions, in the absence of any formal systems and policy that guide the practice of placing MOs at their native places, such practices are perceived as being inconsistent having a negative influence over MOs’ motivation that further affects the performance and turnover. The second problem is that the health department assumes that the MOs always like to work in their native place which may not always be true as reflected by one of the study respondents. And finally, some districts may have many contenders and other districts may have very few if initial placements are solely based on native place.

From the point of view of effective HRM, maintaining a balance between organizational or health department needs which is to have the adequate number and proper distribution of doctors and meeting the main objective of HR policy which is equity and consideration for employees may be very difficult [29]. And in the process of trying to balance between the two, often the department needs may take precedence over individual needs. Hence, we acknowledge that some of the good-intended HRM practices cannot be consistently implemented, and as a result, they sometimes fail to achieve the main objective of HR policies, i.e., equity and consideration for employees. However, we suggest that it is important to address the inequity issues, even if such issues stem from individual perceptions. This is because the intended HR practices aim to improve the performance through the influence they have upon employee attitudes and the perceptions play a very crucial role in influencing the attitudes and behaviors of employees [10].

As the state does not have any formal written policy or guideline for the initial posting of MOs, the study respondents considered the placements to be arbitrary. Hence, one of the plausible implications of not having a clearly laid out policy is that it may allow MOs to use their influence to get placements at desirable locations, as indicated in the study. And it may be argued that in the absence of any policy, the placement systems are more vulnerable to abuse. Research indicates that written rules exhibit more logical designs, higher compliance, and greater effectiveness than unwritten rules [30]. However, having written rules may be a necessary but not sufficient condition for HRH systems to function effectively. This is because even if policies are in place and the current placement systems (how it is intended to be and what is in actual existence) may be perceived positively by the MOs that support fair and equitable staff placements, the translation of policies into action is very important and often the effectiveness of policies depends on the “implementation fidelity” of the relevant HR systems [31]. The systems with weak “implementation fidelity” may lead to systems abuse and corruption [16, 32]. Hence, the need to have written policies but more importantly effective implementation of such policies is important from the HRH perspective.

## Conclusions

The current study focused on exploring the policies, processes, and practices relating to the initial posting for government MOs to fill the research gap identified in this area. Initial posting is a critical aspect of an employment cycle, and the perceptions and experiences of MOs in Gujarat State regarding the processes and practices involved in their initial posting can be crucial in influencing their performance and especially turnover rates and the ability to attract new recruits due to the critical shortage of MOs in the public sector. Effective placement policies are needed, but effective and consistent implementation is needed to gain the trust of the MOs and to achieve staffing that provides equity of access to health services across the state. Striking a balance between HRH policies that satisfy both the needs of the MOs and those of the health department for delivering services throughout the state is challenging. As this was an exploratory study done with small number of MOs, we propose the need for more quantitative studies to understand the scale of problem and more in-depth qualitative studies across the state and nationally to understand the policies and systems relating to the initial posting of MOs and how such policies and systems influence individual behaviors leading to improved or reduced performance and turnovers.
